# Context-Awareness and Biologically Inspired Behaviour Based on Attention Mechanisms for Natural Human-Robot Interaction

**DOI:** 10.3390/biomimetics11050341

**Published:** 2026-05-14

**Authors:** Jesús García-Martínez, Marcos Maroto-Gómez, Arecia Segura-Bencomo, José Carlos Castillo, María Malfaz

**Affiliations:** Systems Engineering and Automation Department, Universidad Carlos III de Madrid, Avenida de la Universidad, 30, 28911 Leganés, Madrid, Spain; jesusgar@ing.uc3m.es (J.G.-M.); marmarot@ing.uc3m.es (M.M.-G.); arsegura@ing.uc3m.es (A.S.-B.); jocastil@ing.uc3m.es (J.C.C.)

**Keywords:** social robots, joint attention, biologically inspired agents, human–robot interaction, context-awareness

## Abstract

The way robots represent the environment, make decisions, and express themselves can positively influence human–robot interaction if they clearly communicate their intentions and needs. To improve human–robot communication, biologically inspired models that mimic human communication skills, including task and scenario-specific contextual information, can facilitate mutual understanding and successful task execution. This paper presents a Context-Awareness and Biologically Inspired Behaviour system to generate a more natural human–robot interaction. The architecture combines sensory information processed by a Joint Attention System that prioritises stimuli based on internal processes with task-related motivations to generate context- and goal-adapted verbal and non-verbal interaction. We evaluate the system through a video-based user study that compares two robots with similar appearances but different behaviours, one using the proposed approach and the other not using the internal state and joint attention mechanisms, to make verbal and non-verbal responses. The results show that participants rated the robot endowed with the proposed system as significantly more sociable, agentic, and animated than the robot without it. Additionally, the robot not showing the responses developed in this work was perceived as more disturbing than the robot integrating the proposed system.

## 1. Introduction

The scenarios in which robots can work are diverse, rich in detail, and require adaptive behaviour to operate successfully [1]. For example, robots have been widely used in education [2,3,4] or health care tasks [5,6,7], where the environment changes constantly, and individuals are sensitive to the robot’s behaviour. For this reason, accurately representing the world’s details is a challenging task that can lead to improved robot performance. However, correctly representing the environment relies heavily on the system’s perception and processing capabilities [8]. Therefore, integrating the robot’s context into its knowledge provides a powerful source of information that facilitates successful task completion and conveys to users a sense of understanding and control over the situation [9,10].

In humans, perceiving and interpreting the environment and our internal signals is essential for making good decisions and behaving [11]. Along this line, robots mimicking humans have shown to produce coherent behaviour and to effectively communicate with people [12]. Robots typically perceive ambient information by combining data from multiple sources, such as cameras, microphones, and tactile sensors [13]. Then, the robot fuses this information with its internal state to construct models that interpret the internal and external stimuli required to complete goals [14]. Nonetheless, the high number of stimuli the robot perceives requires prioritising which ones are more important for the actual task [14].

Some studies have reported that correctly communicating the robot’s intention and exhibiting natural behaviour increase acceptance [15,16] and trust [16] as people can more easily understand the robot’s behaviour. Like in human–human interaction, combining verbal and non-verbal communication is necessary to facilitate such comprehension [17]. However, speech and gesture generation for natural behaviour usually relies on predefined templates/sentences with low scalability or on AI models that require high computational resources, long inference times, and an internet connection [18].

This paper addresses these challenges by presenting and evaluating a Context-Awareness and Biologically Inspired Behaviour (CABIB) system for social robots in human–robot interaction. The main contribution of this work is the integration of a Joint Attention System [14] with a Decision-Making System [19] to generate context-aware behaviours combining deictic speech and goal-oriented gestures. More specifically, CABIB combines: (i) a biologically inspired internal drive model that simulates internal processes and generates motivational states such as hunger, thirst, or boredom, which trigger goal-directed behaviours; (ii) a Joint Attention System that perceives relevant objects in the environment, prioritises them according to the robot’s internal needs, and enables the robot to communicate those needs through spatially grounded verbal and non-verbal behaviours. This combination allows the robot not only to decide what it needs but also to identify relevant objects in its surroundings and communicate those needs in a contextually grounded and embodied manner. The system has been integrated into the Mini social robot [20] to generate real-time responses that adapt to both the robot’s internal state and its external environment. As a result, the robot’s speech can explicitly refer to the objects it needs and their locations in the scene, and its non-verbal behaviour includes goal-directed communicative cues such as looking at and pointing towards relevant objects.

We evaluate our system by conducting a video-based within-subjects study with 133 online participants to analyse whether our approach improves how people perceive the behaviour of our Mini robot. The study compares two videos, each showing a Mini social robot with the same external appearance (they differ only in colour: blue and orange) but displaying different behaviours. While the Orange robot exhibits a biologically inspired behaviour that considers the environmental contextual information both in its speech (e.g., saying the name and position of stimuli it needs) and gestures (e.g., looking and pointing to the stimuli it needs), the Blue one does not consider ambient information involving predefined speech and random movements and does not adapt to the environmental situation or the robot’s internal condition. The study reports the scores for the factors *Sociability*, *Agency*, *Animacy*, and *Disturbance*, which were included in the standard Human–Robot Interaction Evaluation Scale (HRIES) questionnaire [21], control questions about the robots’ context-awareness, and qualitative data obtained through open-ended questions about the participants’ opinions of the robot’s behaviour.

The manuscript continues in Section 2, reviewing works related to this research. Section 3 describes the Mini social robot and the modules of the CABIB system that use attention mechanisms. Then, Section 4 describes the evaluation procedure for analysing people’s opinions about the integration of the proposed system into our robot. Section 5 shows the quantitative and qualitative results of the user study. Section 6 discusses the principal outcomes and presents the system’s limitations. Finally, Section 7 concludes the paper, summarising the main findings and potential future work.

## 2. Related Work

Social robots are increasingly appearing in many scenarios as promising assistive agents. Since they are designed for interacting with people, endowing them with behaviours that users can comprehend and recognise can facilitate the execution of human–robot cooperative tasks. The following sections review related work that investigates how robots represent ambient stimuli and their own internal state to improve their performance. Additionally, the section reviews how robots employ biologically inspired attention mechanisms to enhance their adaptation to contextual conditions. In particular, the system coordinates the robot’s and users’ attention through responses to their social signals or by guiding them towards relevant elements in the environment.

### 2.1. Representation of Ambient and Internal Stimuli Adapted to the Context

A stimulus is typically understood as any sensory input competing for neural representation and behavioural relevance [22]. The perception and structured representation of external stimuli have been extensively studied in robotics as a key principle for adaptive behaviour [23,24,25]. Early and contemporary approaches focus on processing raw sensory data into increasingly abstract representations that enable robots to interpret their environment and act accordingly. For instance, Zaraki et al. [26] proposed a multimodal perception system that integrates information from cameras, microphones, and tactile sensors to generate a high-level description of the environment by fusing heterogeneous sensory cues. This line of research is further reviewed by Mascaro & Chli [27], who analysed how modern robotic architectures are evolving from purely geometric world models towards richer scene representations that incorporate higher-level concepts such as object instances, spatial relations, and places. As the authors highlight, such representations are essential for contextual understanding of the environment.

Moving to social human–robot interaction, we note that Marqués-Villaroya et al. [28] proposed a biologically inspired, multi-component perception architecture that integrates exogenous and endogenous information into a unified 3D world representation. In this framework, ambient stimuli are spatially situated in the environment and used to regulate the robot’s attentional focus. The exogenous layer prioritises salient external stimuli while inhibiting those that have already been attended. In contrast, the endogenous layer [29] modulates attentional responses based on the robot’s current tasks and plans. Similarly, Yi et al. [30] introduced a biologically inspired perception model that represents geometric relations in the environment, enabling robots to situate visual stimuli within a spatial scene and thereby improve the contextual interpretation of ongoing situations.

While ambient perception is essential for understanding the external world, the representation of the robot’s internal state is equally critical for adaptive and meaningful behaviour. Since the pioneering work of Velásquez [31] and Cañamero [32], biologically inspired robotic systems have increasingly incorporated internal variables such as motivation, emotion, and physiological needs. These models draw on psychological and ethological theories to endow robots with internal dynamics that regulate behaviour in a human-like manner [12]. Typically, internal processes are continuously modulated by ambient stimuli, allowing the robot to select actions that are aligned with its goals and internal needs [33].

Following this approach, we developed a controller for social robots [34] that simulates internal processes (such as boredom, sleep, and social needs), which give rise to motivational drives that influence behaviour selection. The perceived environment can amplify or attenuate these motivations; for example, recognising a user may increase the robot’s motivation to engage in play. Although this approach enables autonomous and biologically grounded behaviour, environmental stimuli are treated as discrete entities, and the robot does not explicitly adapt its actions to a structured scene representation. Addressing this challenge at a higher cognitive level, Lapointe [35] proposed a computational framework in which autonomous agent behaviour is regulated by internal drives that are dynamically prioritised according to the situation. In this framework, behaviour emerges from the evaluation of competing internal motivations and actions are selected based on their contribution to satisfying these drives. This view aligns with our work, as behaviour arises from internally regulated motivational states rather than fixed stimulus-response rules. However, while Lapointe focuses on higher-level cognitive drives and semantic reasoning in autonomous agents using Large Language Models (LLMs) to generate context-awareness, our work addresses this problem at the embodied interaction level by combining biologically inspired internal deficits with context-aware perception to generate situated social behaviour in a physical robot.

Beyond internal modelling, several studies explicitly leverage perceptual representations to generate actions adapted to both the task and the interaction context. Aliasghari et al. [36] employed perception to observe users’ behaviours and construct action sequences through imitation during repeated interactions, validating their approach in manipulation tasks such as grasping, pressing, and sliding. Similarly, Chen et al. [37] proposed a model in which the robot anticipates its own mental state based on multimodal sensor data, including speech and emotion cues, to select behaviours that maximise expected benefit. In this way, perception-driven internal modelling enables not only adaptive action selection but also expressive behaviour, such as context-aware speech, which is attuned to the user’s inferred needs.

### 2.2. Attention Coordination in Human-Robot Interaction

Coordinating attention is a fundamental ability in human social interaction. It plays a central role in communication, cooperation, and the establishment of mutual understanding, beginning to develop early in childhood [38]. Through this process, individuals learn to align their focus with others, enabling referential communication and collaborative action. In developmental research, this capacity is referred to as joint attention, defined as the ability to coordinate attention with another individual towards an object or event while being aware that this focus is shared [39].

From the perspective of social robotics, this concept is particularly relevant, as social robots are designed to coexist and interact with people in social environments [40]. Enabling robots to perceive and coordinate attention with users has therefore become an important line of research in human–robot interaction [41]. From a perceptual perspective, *joint attention* relies on stimulus processing. In robotic systems, stimuli may originate from social cues produced by the user (e.g., gaze direction, gestures, or speech [42]) or from the surrounding environment. Environmental stimuli can include low-level features such as brightness [43], colour [44], or motion [45], as well as higher-level elements such as recognised objects or scene events. The focus of attention refers to the stimulus selected for prioritised processing at a given moment, which the robot will then focus on.

The literature distinguishes two main forms of attentional coordination: initiating joint attention (IJA) and responding to joint attention (RJA). IJA involves deliberately directing another agent’s attention towards a selected referent [46]. In this case, the robot generates attentional bids through gaze alternation, pointing, and speech. RJA, in contrast, refers to the ability to follow another individual’s attentional cues, such as gaze shifts or pointing gestures, to establish a shared focus [47]. In robotics, RJA has been implemented through gaze-following and gesture-following mechanisms, often using head pose estimation, saliency detection, or multimodal perception pipelines which also consider verbal cues [48,49,50]. Some works additionally incorporate monitoring mechanisms to verify whether joint attention has been successfully established, sometimes referred to as Ensuring Joint Attention (EJA) [51].

There are different levels of attentional coordination: Dyadic interaction involves mutual engagement between two agents without reference to an external object [52]. In the context of joint attention research, this level is associated with RJA, as it reflects sensitivity to another agent’s attentional cues. More complex coordination introduces a third element into the exchange, allowing attention to alternate between partners and external referents [53]. This configuration aligns with IJA, where one agent actively directs the other towards a specific target. At the highest level, triadic representation involves both agents attending to the same target while recognising the other’s attentional state [54]. This level corresponds to EJA, in which sustained coordination and mutual awareness of the shared referent are maintained over time.

More recent IJA research has often focused on specific populations and structured interventions, particularly children with Autism Spectrum Disorder, using hierarchical prompting strategies and multimodal cue escalation in controlled experimental settings [55]. In these setups, robots typically execute preprogrammed or teleoperated behaviours, such as gaze shifts, pointing, or verbal prompts, to guide the user’s attention towards predefined targets, and attention following is evaluated through external sensing or offline coding [56,57,58,59]. While these approaches have shown effectiveness in therapeutic and training contexts, they are often based on scripted interactions and external supervision rather than autonomous perception-driven behaviours.

In addition, several works have explored joint attention mechanisms in which robots rely on perceptual input to initiate or coordinate interaction with users autonomously. These approaches leverage gaze as a primary social signal [60], enabling the robot to detect user attention and trigger joint activity. For instance, recent studies in human–robot collaboration have demonstrated that gaze cues can be used to initiate joint actions, which allows robots to respond adaptively to the user’s attentional state during shared tasks [61,62]. Similarly, perception-based systems have been proposed to recognise user attention in real time and adjust robot behaviour accordingly, highlighting the importance of integrating attention recognition into interactive scenarios [63,64]. Finally, recent studies have examined how users attribute perceptual access and intentionality to robots when interpreting their gaze as a social cue [65].

When considering works where the robot actively guides the user’s attention (i.e., IJA scenarios), two recurring characteristics are relevant for the present study. First, most IJA systems are scripted or teleoperated, and although the robot performs attention-guiding behaviours, it often does not autonomously select the focus of attention based on perceptual input; the referent is frequently predetermined by the experimental design [56,57,58]. Second, evaluation is commonly based on behavioural measures (e.g., gaze following, reaction time, offline coding) rather than on participants’ direct subjective assessments, where responses are frequently annotated by observers [57,58,59]. These choices are consistent with the need for experimental control. However, they limit the analysis of how users perceive the robot’s behaviour as context-aware, intentional, or socially grounded during attention coordination.

Our research group has developed a biologically inspired Joint Attention System that dynamically analyses and autonomously prioritises visual stimuli during interaction. The system processes both user-related social cues, such as faces, gaze direction, and deictic gestures, and environmental stimuli, including motion, colour, brightness, and objects, in real time. It incorporates attention modulation mechanisms that allow stimuli to be weighted and ranked according to their relevance for attentional coordination, generating a prioritised focus of attention based on the ongoing perceptual input [66].

In a subsequent study, this system was integrated into the Mini social robot [20] to enable autonomous joint attention responses during human–robot interaction. The robot was able to select and respond to the most relevant stimuli detected in the environment in real time. The results showed that the inclusion of autonomous joint attention mechanisms led to improvements in perceived social presence [14], perceived competence and warmth [10], and user engagement [67].

Filling key gaps in the literature on IJA and behaviour generation, the system presented in this paper focuses on autonomous, context-aware attentional coordination. The robot selects its attentional targets based on perceptions of ambient stimuli and internal states. In addition, attention-guiding behaviour is generated through coordinated multimodal cues, including gaze, body movement, and verbal expression. The robot’s attentional priorities are influenced by its internal needs, which affect both its own allocation of attention and the way it guides the user’s attention through variations in expressive behaviour. To the best of our knowledge, prior work has not combined a biologically inspired representation of ambient and internal stimuli with context-aware joint attention mechanisms and evaluated their impact through user-perception measures. The contribution of this paper is to integrate these research directions and analyse how users perceive the inclusion of such mechanisms during human–robot interaction.

## 3. Context-Awareness and Biologically Inspired Behaviour

The software architecture proposed in this manuscript aims to generate context-aware, biologically inspired verbal and non-verbal behaviour using attention mechanisms to enable more natural human–robot interaction in social contexts. This section presents Mini, the social robot used to integrate the system and evaluate this approach with users. We emphasise the contribution of this manuscript: how the robot adapts its perception and actuation capabilities to internal and external conditions. The system modulates perception data to give more importance to those stimuli needed by the robot to complete its goals, generating a behaviour based on attention mechanisms that adapts in real time the robot’s speech (for example, including the name of the object and its position on the scene when requesting something) and gestures (for example, pointing with the arms and looking at the object).

### 3.1. Mini Social Robot

**Figure 1:**
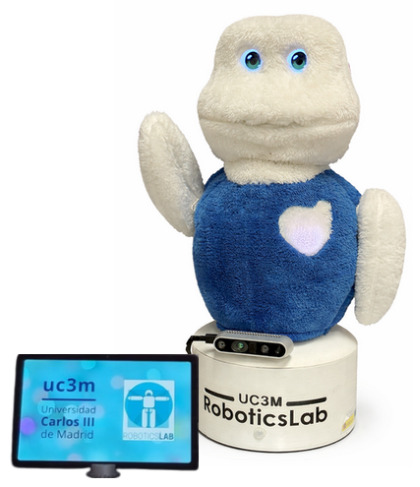
The Mini social robot with its touch screen.

Mini [20] is a desktop social robot conceived to assist people, shown in Figure 1. It was designed by researchers at the University Carlos III of Madrid for various applications, such as health monitoring, cognitive stimulation, and gaming. The robot is used to investigate how to improve human–robot interaction by incorporating biologically inspired mechanisms that make the robot more natural and human-like. Among these mechanisms is the CABIB system presented in this article.

The robot has different sensors for obtaining ambient information. It incorporates a 3D RealSense camera to capture visual stimuli, a noise-cancellation microphone with Automatic Speech Recognition to understand user voice commands, and capacitive touch pads on the shoulders and belly to detect caresses and hits from the user based on contact duration. Mini has a radio-frequency identification (RFID) reader to detect and identify different objects provided by the user. These objects represent food, beverages, and toys that engage the user in the interaction by having them take care of the robot.

Regarding its actuation capabilities, Mini has five degrees of freedom: torso rotation, arm lifting, and neck vertical and horizontal movement. The robot has light indicators in the chest to simulate a heartbeat, the cheeks to simulate blushing, and an LED array in the mouth that lights up when it speaks. It has two animated screens displaying eyes that can be configured online to show different expressions by changing eyelid position, blinking speed and frequency, and pupil size. For verbal communication, it has a stereo speaker with a Text-to-Speech synthesiser that converts text to speech. Finally, the robot has an external touchscreen to display images, photos, and videos during the activities it can execute, and to obtain users’ responses and preferences via touchable menus.

### 3.2. Software Architecture

**Figure 2:**
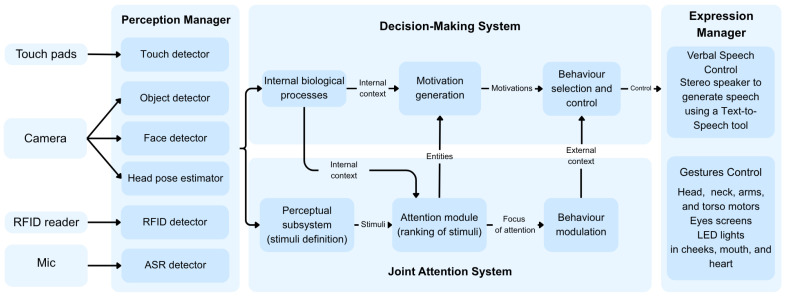
Diagram showing the different modules involved in generating context-aware and biologically inspired behaviour based on attention mechanisms.

The robot’s hardware is controlled by a modular software architecture specifically designed to manage the interaction with its users. This architecture, shown in Figure 2, relies on (i) a Perception Manager, which receives inputs from the sensor and processes them, (ii) a Decision-Making System, which generates biological functions that represent the robot’s internal state and selects the most appropriate behaviour, (iii) a Joint Attention System, which prioritises ambient stimuli based on their importance to the robot’s state and produces verbal and non-verbal responses adapted to the situation the robot experiences, and (iv) an Expression Manager, which commands the actuators and allows multimodal gesture synchronisation. Table 1 shows the contribution of each software module to the system performance.

#### 3.2.1. Perception Manager

The Perception Manager is the module responsible for collecting perceptual data from multiple sensory channels and standardising it into a unified representation that higher-level modules can use. The Perception Manager transforms raw detections into structured key–value messages. Each message contains detector data, including the detector label, the detected feature, confidence information, and, when applicable, spatial coordinates (e.g., 3D position relative to the robot). This standardisation ensures that information originating from different sources, such as vision, audio, touch, and RFID, is represented in a consistent format and published through a standard communication interface.

This module integrates perceptual information from multiple sensory channels. In the visual domain, the system integrates face detection and head pose estimation, implemented using pre-trained OpenVINO models (https://github.com/openvinotoolkit/openvino (accessed on 15 February 2026)). Environmental low-level features include object detection using MobileNet-SSD v2 (https://coral.ai/models/object-detection/ (accessed on 15 February 2026)).

In addition to vision, the Perception Manager processes tactile input from capacitive sensors embedded in the robot and from an external touch screen interface, audio information via an Automatic Speech Recognition system from SemVox (http://www.semvox.de/ (accessed on 15 February 2026)), and object identification via RFID tags.

#### 3.2.2. Decision-Making System

The Decision-Making System is the module that determines the most appropriate behaviour by simulating biologically inspired processes that mimic how human behaviour emerges [19] and by incorporating perceptual information. This module has two specific goals. First, generating a biologically inspired motivational behaviour that drives the robot’s actions, based on simulated internal processes and the stimuli it perceives. Second, sending the robot’s internal state (deficits and actions) to the Joint Attention System to regulate the robot’s attention towards stimuli relevant to its internal context, thereby generating appropriate speech and gestures.

Internal processes emulate human functions such as entertainment or feeding. They elicit motivated behaviour that seeks to (i) endow the robot with the appearance and capabilities of artificial life and (ii) engage the user in the interaction by taking care of the robot. The overall status of the robot’s internal processes represents its internal state, which is influenced by the perception of certain stimuli in the environment. Additionally, the availability (or lack of) certain stimuli motivates the robot to execute actions, such as requesting a resource, rejecting it, or consuming it. These actions have different effects, such as restoring internal processes or communicating to the user what it needs.

The biologically inspired behaviour emerges with the modelling of three internal processes that linearly evolve with time. The model proposed for these processes takes inspiration from how human physiology generates internal deficits to drive behaviour [68]. The processes considered for Mini are feeding, moisturising, and entertainment. All processes have a value ($v_{i}$) at each time step *t* within a predefined range of 0 to 100 for homogeneity. They also have a static ideal value ($i_{i}$) that indicates the optimal state of the process, set at 100 units. At every time step (set to 500 ms), these processes modify their values at a fixed, predefined rate ($r_{i}$), which is set to a linear decay to simplify complex dynamics, deviating from their ideal values (see Equation (1)). For example, feeding gradually decreases, updating its value at each time step and deviating from its ideal value of 100 units, simulating the robot loses nutrients. Similarly, the level of moisturisation decreases over time, simulating how our bodies lose water and electrolytes.

(1) $$v_{i}\left(t\right)=v_{i}\left(t-1\right)-r_{i}$$

Table 2 shows the biological internal processes modelled in our robot and their defining parameters. The variation rates reported in this table were introduced in [19] and determined through an iterative design process that combined conceptual grounding in human physiology with empirical tuning. Specifically, parameters were adjusted through trial-and-error to elicit behaviour activation frequencies that are plausible and interpretable in a human–robot interaction scenario. The goal was to obtain a balanced interaction rhythm in which different needs emerge at different temporal scales, avoiding both excessively frequent and excessively sparse behaviours. For example, feeding-related processes evolve more slowly than hydration or entertainment, leading the robot to request food less frequently than drink or play, which aligns with general human patterns.

The deviation of the robot’s biological processes from their ideal values creates an internal deficit ($d_{i}$) whose pressure increases over time. All deficits range from 0 to 100, as they represent the absolute difference between the ideal value and the current value of the related biological process, as Equation (2) shows. The deficit associated with feeding is hunger; with moisturising, thirst; and with entertainment, boredom. When deficits are high enough (above a threshold of 30 units), the robot’s internal state is poor, motivating the agent to execute behaviours that aim to restore deficits to their optimal state. As the model is updated every 500 ms, these rates produce the following behavioural frequencies: hunger requires approximately 750 s (1500 update steps) to reach an empirically defined threshold of 30 units (that is later used to elicit the corresponding behaviour), thirst around 500 s (1000 steps), and boredom 300 s (600 steps). These values were chosen to ensure that multiple types of behaviours can be observed within a typical interaction session, while maintaining differentiation between processes, avoiding repetitive behaviours.

(2) $$d_{i}\left(t\right)=\left|i_{i}\left(t\right)-v_{i}\left(t\right)\right|$$

Deficits accumulate over time, simulating the robot’s loss of nutrients or that it is becoming bored, leading to the appearance of a related motivation that increases in intensity to drive an associated behaviour. Motivations are psychological, emotional, and biological drives that initiate, guide, and sustain goal-oriented behaviours. Following relevant motivational theories [69], human motivation to execute a particular behaviour is amplified and sometimes depends on the perception of certain stimuli. When a resource is available, the robot can execute a consummatory behaviour to consume it. If unavailable, the robot exhibits appetitive behaviours to make the necessary resource available. For example, the consummatory behaviour of eating depends on food availability and seeing a palatable food amplifies our desire to eat.

Our robot has the motivation to *acquire* what it needs, to *avoid* what it does not need or does not like, and to *consume* what it needs to restore an internal deficit. Each motivation is attached to a behaviour. The robot can *request* something when motivated to acquire it, *reject* something when motivated to avoid it, and *accept* something when motivated to consume it. The accept behaviour, which, conceptually, is eating, drinking, and playing in our scenario, is consummatory, since it directly reduces the robot’s deficit and requires the availability of a resource (food, drink, or toy). Request and reject are appetitive because they do not provide an instantaneous internal benefit to the agent. Table 3 shows the motivations that drive the robot’s behaviour based on its internal and external state, including the type of object involved, the resource conditions, and the effects of each behaviour.

An example of how this module works is the control of the robot’s entertainment. After some time without playing, the robot becomes bored because its entertainment process gradually decreases, deviating from its ideal value. In this situation, the robot will be motivated to acquire a toy and will verbally request it, as it is the stimulus it needs. If the user gives a toy (for example, a teddy bear), the robot will accept it, simulating play, and the boredom will disappear since the entertainment process has been restored. Another example is when the user gives the robot an object that it does not need. For example, receiving food when not hungry will drive the robot to reject it.

These patterns equally occur with the other biological processes. To avoid shallow, low-intensity motivations driving a robot’s behaviour, we have empirically defined that a deficit must have at least 30 units to elicit a behaviour. Additionally, to prevent the robot from repeating the same sentences (for example, continuously requesting food when hungry), we have added a timeout to prevent the robot from executing the same behaviour.

The following section describes how the Joint Attention System generates verbal and non-verbal expressions adapted to the context, based on external stimuli from the Perception Manager and internal deficits from the Biologically Inspired Motivational Behaviour System.

#### 3.2.3. Joint Attention System

The Joint Attention System [66] orchestrates the robot’s attention between the user and the environment. It regulates the robot’s focus of attention in real time and translates attentional decisions into coordinated speech and gestures during human–robot interaction. The system is designed as a three-layered framework—Perception, Attention, and Behaviour modulation—which draws inspiration from human psychobiological principles to achieve natural social interaction [70,71]. Through this architecture, the robot can proactively and autonomously guide the user’s attention towards elements that become relevant within the interaction, using coordinated gaze, speech, and pointing behaviours to establish joint attention.

The Perceptual Subsystem receives standardised detections from the visual channel of the Perception Manager and transforms them into internal attentional units referred to as stimulus. Each stimulus, denoted as *S*, is defined as a multi-dimensional vector (see Equation (3)):

(3) S={P,λ,D,κ},

where P=(x,y,z) represents the three-dimensional spatial position in centimetres relative to the robot’s reference frame, λ denotes the initial saliency weight that depends on the stimulus type and its contextual relevance ranging from [0.5,1], *D* is a feature vector that includes semantic information such as the label that identifies the stimulus, the corresponding attribute value, and a confidence score that measures how sure is the detector about the stimulus identification. For example, the object detector may detect a stimulus of type “object” with an attribute value “teddy bear” and a confidence score of 0.9. The final component, κ, represents an inactivity parameter that monitors the duration since the stimulus was last detected.

The assignment of λ values, shown in Table 4, represents the contextual relevance of each stimulus type and was guided by both empirical testing and insights from the cognitive science literature on attentional selection. In this work, the highest value is assigned to the head pose detector (λ = 1.0) when the user is not facing the robot, as it provides a strong cue about the user’s attentional state. When the user is looking at the robot, the value of this detector is set to 0. This prioritisation is consistent with findings such as those of Frischen et al. [72], which emphasise the central role of gaze following in attentional processes. The face detector is assigned an intermediate value (λ = 0.7), as it enables the robot to detect the user and maintain face-to-face interaction. Ferreira et al. [73] highlight that faces are highly salient stimuli that naturally attract human attention. Finally, objects, which are considered environmental stimuli in our system, are assigned a default baseline value (λ = 0.5), enabling higher-level decision-making processes to modulate their relevance according to the robot’s internal state, goals, or task requirements. All intensity values can be modified in real time, either globally (per detector) or individually (per object label or class).

To manage environmental complexity, the Attention submodule of the Joint Attention System groups stimuli into higher-level perceptual “entities” inspired by Gestalt principles of proximity and continuity [74]. Spatially and temporally coherent stimuli are aggregated into unified representations (e.g., a user entity composed of face and head pose; or an object entity composed of visual features and spatial localisation). Each entity *E* is assigned a dynamic priority weight w(t) that captures the evolution of that entity’s priority over time. This weight is modulated by attentional mechanisms inspired by the Inhibition of Return principle [75], which promotes the exploration of new stimuli by progressively reducing the priority of entities that remain in the scene for prolonged periods. However, this weight can also increase under certain conditions, allowing relevant entities to regain priority over time. The overall intensity of an entity is computed as shown in Equation (4):

(4) $$E_{I}=\sum_{i=1}^{n}w\left(t\right)\cdot \lambda_{i}$$

where w(t) represents the dynamic priority weight of that entity and $\lambda_{i}$ denotes the base intensity assigned to the *i*-th stimulus belonging to entity *E*. In this formulation, an entity is defined as a group of stimuli, and its global intensity is computed as the sum of the base intensities of its stimuli modulated by the dynamic entity weight. The resulting entity intensity $E_{I}$ is empirically bounded within the range [0,3].

At every time step, the dynamic weight w(t) of all entities present in the scene is progressively reduced following a predefined decay rate τ=0.2. This decay prevents the same entity from dominating the attentional system for long periods and encourages redistribution of attention across the environment. In addition to this decay mechanism, a set of empirically defined modulation rules allows certain entities to increase their priority. Specifically, entities corresponding to the user closest to the robot, or to users who establish mutual gaze with the robot, receive an increase in their dynamic weight.

These rules are additive. For example, in a scenario with multiple users, a user who is both closer to the robot and looking at it will have higher priority than a user who is farther away or only looking at the robot. In addition, internal robot states or task-dependent configurations (e.g., prioritising objects when an internal need is active) can modify the attentional selection process.

**Figure 3:**
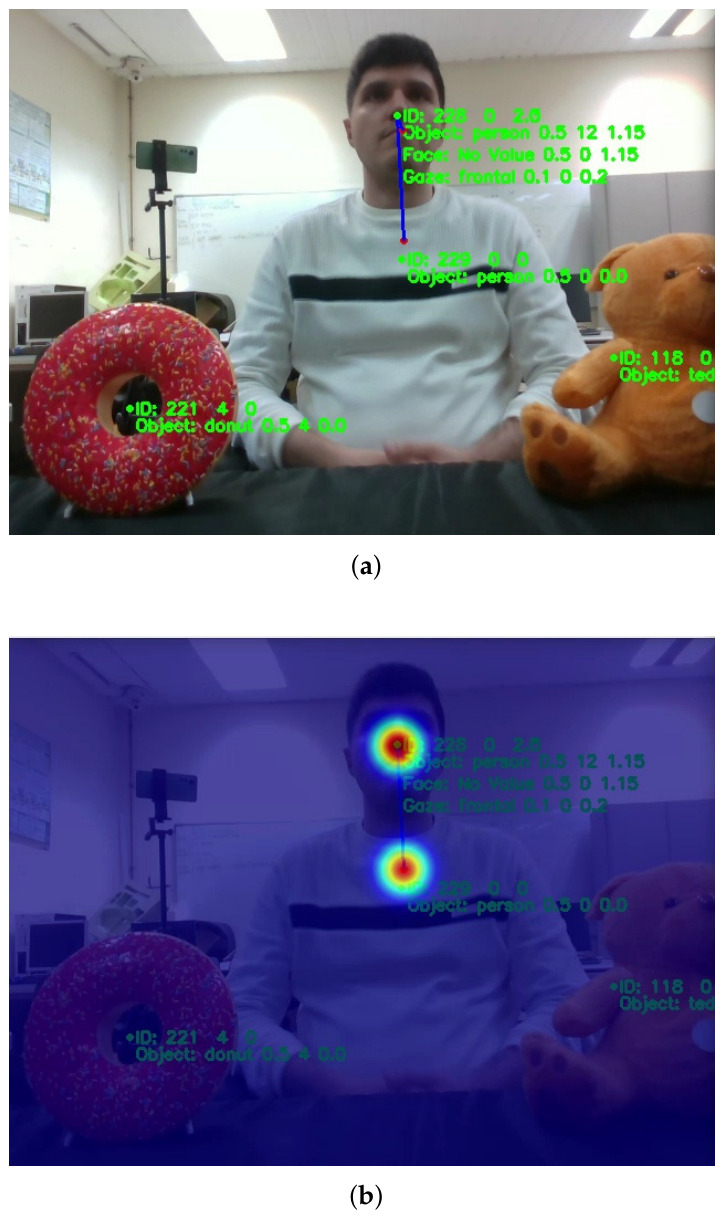
Visual output of the Joint Attention System in a scenario where one user and two objects are present in the scene. (**a**) Visual representation of the clustered stimuli: individual detections are aggregated into entities, each assigned a unique ID. (**b**) Saliency map highlighting regions of interest, with the focus of attention encoded in warmer colours.

The entity with the highest $E_{I}$ value is designated as the current focus of attention. Within that entity, the stimulus with the highest base intensity λ is selected as the effective attentional target. If the selected stimulus contains spatial information, its three-dimensional coordinates are extracted and used as the motor reference for actuation. Figure 3 illustrates the Joint Attention System output in a scene where one user and two objects (a doughnut and a teddy bear) are present within the robot’s field of view. The output incorporates perception information divided into two groups: (i) estimated information from each entity, such as its type (e.g., object or person) and complementary data, which includes gaze direction for the user, as shown in Figure 3a; (ii) the priority assigned to each entity, represented as a heat map in Figure 3b. The Behavioural Modulator translates the selected focus of attention into behaviour-modulation signals sent to the Decision-Making System to adapt the robot’s behaviour. For example, if a stimulus is part of the request, this submodule provides its position in the scene and its semantic content to adapt the speech.

A defining characteristic of the present system is its integration with the Decision-Making System and the Expression Manager to produce contextual behaviours. All detected objects are semantically mapped to categories associated with the robot’s internal processes, namely feeding, moisturising, and entertainment. Internal deficits are transmitted to the Joint Attention System, where they act as top-down modulators of attentional weights. When a deficit becomes behaviourally relevant, the Attention Module increases the priority of entities corresponding to objects linked to that need. For example, if the robot experiences a high “hunger” deficit (as described in Section 3.2.2), the saliency of an object identified as “food” is significantly boosted. This allows the robot to initiate an IJA sequence, such as performing a gaze shift towards a doughnut on a table while simultaneously stating: “I am hungry; please give me the doughnut located to your left.” This integration allows the robot’s attention to be not only reactive but also driven by its internal state and goals.

Through this architecture, attentional selection emerges from the interaction between bottom-up perceptual input, socially driven cues, and internally generated motivational signals. As a result, the robot can both respond to user attention and proactively guide attention to contextually relevant elements.

#### 3.2.4. Expression Manager

The Expression Manager controls the robot’s speech and gestures through two submodules: Verbal Speech Control and Gesture Control. The robot’s Verbal Speech Control module generates utterances adapted to the attentional and motivational context. It is implemented as a keyword-replacement system in which sentence templates are dynamically completed based on the selected object category (e.g., “the teddy bear”) and its spatial relation to the user (e.g., “to your left”, “in front of me”). In RJA scenarios, the robot may acknowledge the user’s referential act. In IJA scenarios, it produces explicit verbal cues aligned with the selected focus of attention.

The Gestures Control follows a strategy inspired by the human vestibular system and eye–head coordination mechanisms [76]. For each selected focus of attention, the controller computes the joint positions and angles required to align the robot’s gaze and body orientation with the target. The movement sequence follows a vestibular-inspired coordination pattern. First, the eyes rapidly reorient towards the target until reaching their mechanical limits or the desired fixation point. Subsequently, the head and neck rotate to reduce ocular deviation, while the eyes compensate in the opposite direction to stabilise the gaze, approximating human saccadic coordination and maintaining fixation during motion. Finally, if required, the torso reorients to align the upper body with the attentional target once the head–neck system approaches its range limits or the target lies outside its effective workspace. In IJA scenarios involving referential actions, the controller can also activate the arm joints to perform a pointing gesture aligned with the selected object.

## 4. Evaluation

This section describes the evaluation procedure for assessing users’ perceptions of the robots’ behaviour. We conducted a video-based within-subjects user study to compare two identical robots in appearance (except for their colour) but different in behaviour, to analyse whether people perceive such differences. The behaviour difference depends on the use of the CABIB system, which is based on attention mechanisms. The evaluation is centred on object-request interactions in which the robot initiates joint attention behaviours driven by its internal motivational state and the perceived context. The conditions evaluated in this study are:

- **Robot with Context-Aware Attention (RC):** The robot integrates the biologically inspired Decision-Making System with the Joint Attention System. The behaviour is driven by internal deficits (e.g., hunger, thirst, or boredom), which generate goal-directed motivations such as acquiring, consuming, or avoiding objects. The robot also perceives and localises relevant objects in the environment through vision, prioritises them according to its internal needs, and grounds its behaviour in this contextual information. The robot’s speech explicitly refers to the object it needs and its location in the scene. Its non-verbal behaviour includes context-grounded communicative cues such as looking at and pointing towards relevant objects.
- **Robot without Context-Aware Attention (RN):** The robot uses the same biologically inspired Decision-Making System. It therefore exhibits the same internal deficits and motivational behaviours (request, accept, and reject) as the RC condition. However, this condition does not include the Joint Attention System and therefore lacks visual perception and attentional selection of environmental stimuli. Object perception is limited to RFID detection when the user physically presents an object. Consequently, although the robot can still generate the intention to request an object, it cannot identify or refer to a specific object in the environment. Its speech is expressed through predefined generic utterances, and its movements are non-directed. To avoid Mini from being static all the time, the robot performs random movements that do not convey attentional or communicative intent.

### 4.1. Hypotheses

The premise of this study is that participants can observe changes in the robots under the RC and RN conditions and that these changes positively influence various factors. Based on this focus, the hypotheses we formulate are:

**Hypothesis** **1.** *Participants will perceive the robot showing the context-awareness attention system as more sociable than the robot that is not aware of its surroundings, since its speech and gestures are richer and more focused on the current situation.*

**Hypothesis** **2.** *Participants will rate the robot with the CABIB system as significantly more intelligent and intentional than the robot without the system, since this robot employs detailed ambient information to interact with the user.*

**Hypothesis** **3.** *Participants will perceive the robot with the CABIB system as more animated and natural since it has more human-like expressiveness.*

**Hypothesis** **4.** *We do not expect participants to report differences in disturbance since the external appearance of both robots is the same, and there are no negative attitudes towards the robot during the experiment.*

### 4.2. Participants

One hundred thirty-three people participated in this study, 67 of whom were men and 66 were women, with ages from 16 to 84 years old (μ=39.43,σ=15.99). All participants voluntarily gave their explicit consent, in accordance with the Data Protection Policies accepted by the University Carlos III Committee. The participants were recruited through advertisements posted on social media and messaging applications, as well as distribution lists available at the University Carlos III of Madrid. This study was exempted from Ethics Approval since it does not involve clinical studies.

More than 74% of the participants (99 vs. 34) had never interacted with the Mini robot or watched videos about it. We collected participants’ technology and robotics knowledge for further analysis using a five-point scale from very low to very high. 10 participants indicated very low technology level, 28 low level, 48 moderate level, 26 high level, and 21 very high. This distribution suggests that our sample has a moderate level of technology. Regarding robotics knowledge, 50 participants indicated a very low technology level, 37 a low level, 21 a moderate level, 10 a high level, and 15 a very high level. Therefore, most people were naive about robotics, and some claimed to be experts.

### 4.3. Procedure and Scene

**Figure 4:**
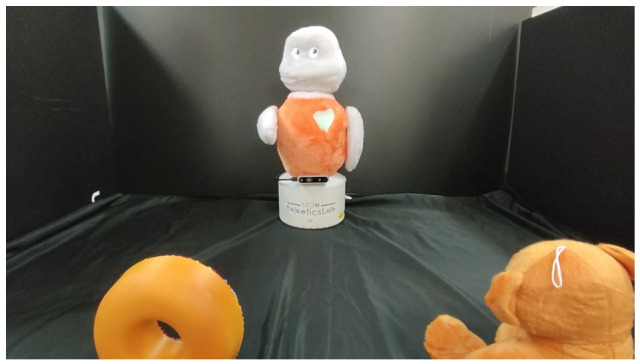
First-person scene for Scenario 3 presented with the two objects (a doughnut on the left and a teddy bear on the right) on the table and the robot in front of the user.

The evaluation procedure consisted of spreading an online survey intended for the general audience. Participants had to watch two videos and then complete a form with quantitative and qualitative questions asking for their opinions on the behaviour exhibited by both robots. One of the videos showed a Blue robot (condition RN) and the other an Orange robot (condition RC). Both have the same appearance (except for the colour of their external plushy cover) and differ in the behaviour they exhibit. The videos are in first person from the participant’s point of view. In the scene shown in Figure 4, there is a table with the Orange robot at the centre back and two objects situated at both sides of the table.

Each video contained three scenarios with the same scene. The three scenarios are the same in both conditions. They show a robot with an internal need (hunger, thirst, or boredom) requesting something from the user, and the user picking up an object from the table and giving it to the robot. We used a teddy bear, a water bottle, and a doughnut as the objects the robot needs, and the user must hand them to the robot. These objects are all equipped with an RFID tag that the robot can detect with the reader mounted on its belly. The scene’s background is black to avoid distracting participants with other stimuli.

In Scenario 1, the robot is at the back, with a teddy bear on the left and a water bottle on the right. The robot is bored, so it asks the user for something to play with. The user takes the teddy bear (equipped with an RFID tag) and rubs it on the robot’s belly. After this, the robot reacts to this event, saying thank you to the user. Scenario 2 shows the robot at the back with a doughnut on the left and a water bottle on the right. This time, the robot is thirsty, so it asks the user for the bottle. The user picks up the bottle and gives it to the robot, which reacts to the event. Finally, Scenario 3 shows the same situation, but with a hungry robot, the doughnut on the left and the teddy bear on the right.

The difference in the videos is the robot’s behaviour. The Blue robot behaves without the CABIB system (condition RN). The robot is not aware of the external environment, so its verbal responses are generic (e.g., it requests a toy even though a teddy bear is on the table), and its non-verbal gestures do not consider environmental information when looking or pointing to the objects needed. In contrast, the Orange robot behaves using the CABIB system presented in this manuscript (condition RC). It is autonomously capable of selecting, from the available objects in the scene, the one that best satisfies its current internal need. Since it is aware of the scene and can perceive it, its speech includes the name of the objects it needs and their position on the table. Additionally, when requesting the object, the robot looks at it and points to it, emphasising its need for it.

### 4.4. Form Content

The experiment was conducted using an online survey on Google Forms. The form was divided into four sections: cover page with consent agreement, personal data collection, videos of the two robots (blue and orange), and a post-experiment questionnaire. Next, we explain the content of each section. The questions on each page, except the cover page, were randomised. This includes the order of appearance of the two videos, which was randomised to avoid the situation in which watching the first robot affects perception of the second.

#### 4.4.1. Cover with Consent Form

The cover page described the aim of the experiment, its approximate duration (about 10 min), and requested users’ explicit consent to participate. This page included two checkboxes whose content was approved by the Data Policies Committee of the University Carlos III of Madrid: participants could indicate whether they authorised the publication of their previously pseudonymised data, if they were informed via email, and whether their data can be used for other research projects if necessary. Finally, the cover included contact details and their rights regarding the execution of the experiment. By pressing Continue and accepting at least the first policy, they could proceed to complete the rest of the questionnaire.

#### 4.4.2. Personal Data

The second page collected personal information from the participants for further detailed statistical analysis. We collected their gender (male, female, other), age (open-text question limited to a number from 16 to 99), technology and robotics knowledge (on a five-point scale), and whether participants had previously interacted with the Mini robot, either face-to-face or virtually (Yes or No). These questions had to be filled in. All responses were pseudonymised before analysis, and participants were referenced using non-identifiable IDs (e.g., ID = 15), ensuring that no personal data could be linked to individual participants.

#### 4.4.3. Videos

The survey continued showing two videos labelled as *Blue robot* and *Orange robot*. Each video showed three numbered scenarios: the robot having an internal need, the user picking up an object from the table in front of the robot, and then rubbing the object on the robot’s belly to be detected by the RFID sensor. The Blue and Orange robot videos differed in the speech and gestures displayed by the robots, which were adjusted to each condition evaluated. The playlist https://youtube.com/playlist?list=PLXNPQDsfy0lLcwCiPxohLRfToerbyAQzV&si=yzitGcExxsaItL57 (accessed on 19 February 2026) contains the videos used for the evaluation, both in English and Spanish, and a video showing what the robot perceived during the interaction. Participants could watch the videos as many times as desired.

#### 4.4.4. Post-Experiment Questionnaire

Participants could watch the videos as many times as desired. We designed a post-experimental questionnaire that combines quantitative and qualitative questions to gather the users’ impressions. For the quantitative analysis, we used the standard Human–Robot Interaction Evaluation Scale (HRIES) [21]. This questionnaire measures the participants’ opinions towards the robot factors *Sociability*, *Agency*, *Animacy*, and *Disturbance*. Each factor consists of 4 items, each scored on a seven-point Likert scale. *Sociability* represents the ability of an individual or a group of individuals to evolve in society. *Agency* groups items that define unique human capabilities such as intelligence. *Animacy* includes items that suggest human characteristics for non-human agents. Finally, *Disturbance* represents the negative perception of robots, characterised by uncomfortable feelings.

In addition to the HRIES questionnaire, we included three qualitative queries. Participants were asked the following questions: *Have you noticed if any of the robots could perceive their surroundings?*, *Have you felt if any of the robots looked or pointed to the objects they wanted or needed?*, and *Have you felt if any of the robots is aware of its own state and environment?*. Participants could answer these three questions with the options None of the robots, Both robots, Blue robot, or Orange robot. All the quantitative questions had to be completed to proceed to the next question.

Furthermore, we allowed participants to discuss their feelings through three open-ended questions. These questions were not mandatory. The questions selected were: *Briefly define the behaviour of the Orange robot*, *Briefly define the behaviour of the Blue robot*, and *Commentaries and Suggestions*.

## 5. Results

The results of the user study are divided into two sections. First, we present participants’ scores of the two robots using the HRIES questionnaire and three questions about whether they perceived specific responses from the robots. Second, we analyse the qualitative data collected through open-text questions administered to the participants.

### 5.1. Quantitative Results

The HRIES questionnaire assesses users’ opinions towards the robot *Sociability*, *Agency*, *Animacy*, and *Disturbance*. We conducted a statistical analysis in SPSS Statistics v. 31 (IBM) to compare the two robots. Descriptive statistics for each HRIES factor by robot condition are presented in Table 5. We report medians, interquartile ranges (IQRs), means, and standard deviations (SDs), along with significance results and effect sizes. Kolmogorov-Smirnov normality tests (N>50 participants) revealed (p<0.05 for all but Animacy, p=0.200). These results justify the use of non-parametric methods in the following analyses.

Given the within-subject design and the non-normality of the data, we used the Wilcoxon signed-rank test to compare participant evaluations across robot conditions for all four factors and reported the effect size using Rosenthal’s r [77]. The statistical tests were conducted at a significance level of α=0.05, with a Bonferroni correction to control the family-wise error rate. In particular, the four dependent variables measured for the robot correspond to related dimensions of robot attributes, forming a coherent family of tests. Therefore, the adjusted significance threshold was set to α=0.05/4=0.0125. This decision ensures that the probability of false positives is controlled across the full set of comparisons, providing stronger support for statistically significant findings.

A sensitivity power analysis was conducted using GPower 3.1.9.7 for the within-subjects design. We used a paired-samples test, two-tailed α=0.0125, statistical power = 0.80, and N=133 paired observations. The analysis indicated that the study was powered to detect a minimum effect size of dz=0.293. This analysis applies to the four HRIES comparisons, as they shared the same within-subjects design, sample size, and Bonferroni-adjusted significance threshold.

**Figure 5:**
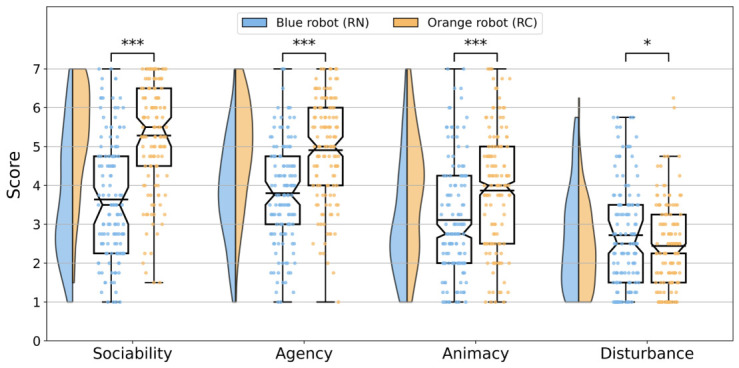
Boxplots with distributions showing significant differences for the four factors analyses: Sociability, Agency, Animacy, and Disturbance. The number of asterisks (*) indicate the level of statistical significance: “***” indicates very significant $p_{\mathrm{value}}<0.001$ and “*” indicates subtly significant $p_{\mathrm{value}}<0.05$.

The results, shown in Figure 5, indicate statistically significant differences between the Blue RN and Orange RC robots for all factors. Participants rated the Orange robot significantly higher than the Blue robot in Sociability (Z=−7.84, p<0.001, r=0.68), Agency (Z=−6.20, p<0.001, r=0.54), and Animacy (Z=−7.96, p<0.001, r=0.69), indicating large effect sizes. In contrast, Disturbance was slightly higher for the Blue robot (Z=2.82, p=0.005, r=0.24), representing a medium effect size.

All *p*-values remained significant after applying the Bonferroni correction for multiple comparisons (adjusted $\alpha =\frac{0.05}{4}=0.0125$), confirming the robustness of the observed effects against Type I error. The most significant effects were observed in Sociability, suggesting that participants found the Orange robot more sociable in terms of likeability and warmth than the Blue robot. Similarly, participants found the Orange robot to be more agentic, suggesting it is more intentional and intelligent than the Blue robot. Finally, the significant results obtained for Animacy indicate that the Orange robot looks more human-like. The effect on Disturbance, while statistically significant, was smaller than for the other factors, suggesting that the Orange robot was perceived as less creepy and weird.

Table 6 shows the distributions of responses to the three questions asked of participants about whether they perceived specific robot responses during the experiment. To the question *Have you noticed any of the robots could perceive their surroundings?*, 96 out of 133 people (72%) answered they perceive the Orange robot to have this capacity, 20 (15%) indicated that both, 10 participants that none of the robot (8%), and 6 that only the Blue robot (5%). For the second question, asking *Have you felt that any of the robots looked or pointed to the objects they want or need?*, 88 people (66%) indicated that only the Orange robot, 28 that both (21%), 11 that none (8%), and only 6 participants out of 133 (3%) perceived it in the Blue robot. Finally, the answers to the question *Have you felt that any of the robots is aware of its own state and environment?* reported similar patterns. 108 participants (81%) indicated that only the Orange robot, 9 that both (7%), 4 that none (<3%), and 12 people (9%) perceived such capacity in the Blue robot.

### 5.2. Qualitative Results

In addition to the HRIES scores and the three questions about behaviours perceived in the robot, we analysed the optional open-text responses collected at the end of the questionnaire. Participants were asked another three questions: (Q1) “Briefly describe the behaviour of the Orange robot”; (Q2) “Briefly describe the behaviour of the Blue robot”; (Q3) “Suggestions and comments”. As responses were not mandatory, several participants left one or more questions unanswered. After removing empty or non-informative entries, 102 valid responses were retained for Q1 and Q2, and 37 for Q3. An inductive thematic analysis was then performed on the remaining text [78]. Two researchers independently reviewed the Spanish responses, extracted salient keywords, and iteratively grouped them into recurring themes and sub-themes. Inter-rater reliability was assessed using Cohen’s κ for each question independently. Agreement was substantial for Q1 (κ=0.61), moderate for Q2 (κ=0.43), and strong for Q3 (κ=0.84), based on support-weighted averages across codes. These results indicate a generally positive level of consistency between researchers, particularly for Q1 and Q3. Disagreements were resolved through discussion until a consensus was reached. Representative excerpts (translated to English for reporting) were selected to illustrate each theme.

For responses regarding Q1, the most frequently identified theme associated with the Orange robot was *social tone and politeness*, identified in 66 responses. Participants often described the Orange robot as friendly, close, cordial, or grateful (e.g., “friendly”, ID = 8; “close”, ID = 11; “polite and friendly”, ID = 76), and several responses associated this with increased perceived confidence or comfort (e.g., “the Orange robot gives more confidence”, ID = 11).

The second major theme concerned *specificity in requests*, mentioned in 48 responses, where participants highlighted that the robot did not only state an internal need but also linked it to concrete objects and their spatial location (e.g., “it looks at and points to the object it wants and identifies left and right”, ID = 22; “it gives clear indications of where the object is with respect to the person”, ID = 38).

Closely related to this subject, the Orange robot was characterised as *context-aware* and *interaction-oriented*. A total of 44 responses explicitly referred to the robot as being aware of, or able to analyse, its surroundings (e.g., “more aware of what it has around it”, ID = 1; “it analyses the environment”, ID = 34). Related to this, 11 participants frequently mentioned *deictic and embodied cues* (gaze/pointing), describing the behaviour as more proactive and informative (e.g., “it explains and clearly points to the object it wants”, ID = 9; “it looks at them and points to them”, ID = 78). While most comments were positive, a smaller set of responses (3 participants) noted that the orange behaviour could become overly explicit or insistent depending on the context (e.g., “great for beginners, but too much becomes a bit heavy and cuts the rhythm”, ID = 9), suggesting a perceived trade-off between clarity and interaction pacing.

Concerning Q2, the Blue robot was commonly described as *vague in communication*, present in 44 responses: the robot was perceived as stating needs in a general way and leaving the user to infer what object to provide (e.g., “it gives a clue and leaves the user to deduce what it wants”, ID = 9; “it only states a desire like eating but neither points nor names the object”, ID = 92). An equally frequent theme (44 responses) referred to *coldness or reduced social warmth*. Many participants used terms such as “cold”, “dry”, “robotic”, “rude”, or “distant” (e.g., “dry”, ID = 8; “robotic”, ID = 26; “rude and unpleasant”, ID = 76).

A further theme concerned *dependency or passivity*, mentioned in 25 responses. Participants described the Blue robot as relying on the human to resolve the situation (e.g., “more infantile and dependent on the human”, ID = 57; “it says what is happening and waits for someone to solve it”, ID = 88). In 15 responses, the Blue robot was described as *less grounded in the environment* and *less socially attuned*, with participants noting that the robot seemed unaware of surrounding objects or did not reference them explicitly (e.g., “as if it cannot see the environment”, ID = 34; “it does not indicate where things are nor point to them”, ID = 64).

A related but less frequent sub-theme involved *static or non-committal movements*, present in 9 responses. Participants described the behaviour as static or composed of broad, non-committal movements (e.g., “too static, it looks everywhere but does not fix its gaze”, ID = 16; “random head movements”, ID = 56), which was sometimes interpreted as confusion or detachment (e.g., “more lost and isolated compared to the orange”, ID = 82).

Finally, a minority of participants (3 responses) perceived the behaviour of the Blue robot as more *concise* or potentially more *practical* in scenarios where object availability is uncertain, noting that explicit requests may presuppose the presence of a specific resource (e.g., ID = 88). Overall, the dominant interpretation was that the Blue robot communicated needs with fewer contextual cues, which reduced clarity and social interpretability for many participants.

With respect to suggestions and comments (Q3), the most frequent theme concerned *non-verbal expressivity and embodiment*, which appeared in 9 responses. These included requests for improvements in synchrony and embodiment, such as mouth movement while speaking and improving visual expression (e.g., “it does not move its mouth enough when speaking, which makes it strange”, ID = 21; “The feeling of interaction could be improved by expanding the capacity for contact and visual expression”, ID = 103).

The second most frequent theme was related to *verbal naturalness*, mentioned in 7 responses. Participants noted the mechanical quality of the voice and requested a friendlier or more natural tone (e.g., “the voice is very mechanical; it should be sweeter”, ID = 29; “both have a robotic voice”, ID = 34).

Two additional themes were equally represented (6 responses each). The first, coded as *observations*, includes general remarks about the study or the perceived maturity of the behaviours (e.g., “it seemed like a curious and entertaining experiment”, ID = 50; “both have a lot of room for improvement, but it is evident that the orange is one step ahead of the blue”, ID = 101). The second concerned practical *issues* related to the study material, such as potential confusion in the form, particularly regarding the ordering of orange/blue options relative to video order (ID = 100), and limitations of the video display interface (ID = 122). Lastly, *interaction design trade-off* was mentioned in 5 responses, suggesting that the Orange robot is beneficial for users who may require more guidance, while a less explicit style could be preferable in other contexts (e.g., ID = 6).

## 6. Discussion

The results obtained in this study support the general view of the HRI community [36,79,80] that modelling the communicative skills of social robots in a human-like manner, in terms of naturalism and comprehension, might have positive effects on human–robot communication. In our study, this is reflected through the discussion of the envisioned hypothesis.

We first hypothesised that the robot with the CABIB system would be perceived as more sociable than a robot using predefined speech and random gestures (Hypothesis 1). The results support this hypothesis, as the Orange robot obtained significantly higher scores than the Blue robot in the Sociability dimension of the HRIES questionnaire. This suggests that integrating context-aware verbal and non-verbal behaviours, such as referring to relevant objects and directing gaze and gestures towards them, improves the perception of the robot as a socially engaging interaction partner. In addition, the observed effect size was large and clearly above the minimum detectable threshold, indicating that the study had sufficient sensitivity to capture this difference between conditions reliably. Nevertheless, since the evaluation was conducted through videos rather than on-site interactions, further in-person experiments are necessary to determine whether social behaviours are similarly perceived.

The Hypothesis 2 stated that the Orange robot would be perceived as more intelligent than the Blue robot. This assumption was measured using the Agency factor from the HRIES questionnaire. The results reported that the Orange robot is perceived significantly more agentic than the Blue robot, thereby supporting the hypothesis. Additionally, people identified that the Orange robot was more intentional and aware of its environment, as indicated by the three questions about the robot’s behaviour.

The Hypothesis 3 evaluated whether the Orange robot is perceived as more animated and natural than the Blue robot. The results suggest that our initial hypothesis is correct, as the scores on the Animacy factor were significantly higher for the Orange robot, indicating that participants perceived it as more human-like and natural. Therefore, during human–robot interaction, people may prefer robots that resemble human behaviour. Additionally, although the HRIES questionnaire does not directly measure the interaction, the high scores on the Sociability and Animacy factors suggest that the interaction is also perceived as more natural.

Finally, the Hypothesis 4 yielded unexpected results. We hypothesised that participants would not perceive differences in Disturbance between the two robots since their external appearance is identical; the HRIES measures items such as weird or scary, and the robot behaviours are not consistent with this kind of response. However, the results reported that the Blue robot was perceived as more disturbing than the Orange robot. In this case, the observed effect size for Disturbance (r = 0.24) corresponds to a small-to-moderate effect, which is closer to the minimum detectable threshold. Nevertheless, the result reached statistical significance after Bonferroni correction, suggesting that the sample size was sufficient to detect even relatively subtle differences between conditions. A possible reason for this result is that people perceived the Blue robot with random movements and gaze as weird and not responsive.

Regarding the results for the three questions asking whether the robots were aware of their surroundings, looked or pointed to the objects they needed, and were aware of their internal state and environment, they are very similar. Most people perceived that the Orange robot was aware of its surroundings (87%), whereas only 20% identified such behaviour in the Blue robot. 87% of the participants also perceived that the Orange robot looked at and pointed to the objects it needed in the scene, against a 24% for the Blue robot. This subtle increase in the second question for the Blue robot might be due to the robot’s random movements, which at some point led participants to believe the robot was looking or pointing to the objects. Finally, for the third question, most people (88%) identified that the Orange robot was aware of its internal state and the environment, while only 17% identified this capacity in the Blue robot.

Our user study compares the behaviour of two robots that appear identical (except for their colour) but differ in behaviour. Therefore, the HRIES results primarily capture relative differences between the two robots rather than providing an absolute benchmark for other human–robot interaction scenarios. To contextualise these findings, we compare our results with previous studies using the Mini robot. In a prior study where Mini expressed preferences and emotional reactions to RFID-tagged objects [81], participants reported lower Sociability (5.28 in this study vs. 5.05 in the other) and higher Agency (3.87 in this study vs. 4.67 in the other). Similarly, in a gesture-generation study [17], Mini achieved higher scores in Sociability (5.76 vs. 5.28), Agency (5.41 vs. 3.87), and Animacy (5.05 vs. 4.91), while being perceived as less disturbing (<2 vs. 2.44). These differences suggest that the interaction paradigm influences users’ perceptions of the robot’s behaviour.

In the present study, the request-giving task requires the robot to exhibit proactive and goal-directed behaviour, explicitly communicating its internal needs and intentions. This increased demand for autonomy and intention expression may expose limitations in the current system, particularly in conveying clear, natural, goal-directed behaviour, which could explain the lower perceived Agency compared to prior work. In contrast, previous studies relied more on expressive behaviours such as gestures and emotional reactions, which may be more immediately interpretable by users and therefore enhance perceived Sociability and Animacy. Finally, the video-based evaluation used in this study, rather than in-person interaction in prior work, may have heightened participants’ sensitivity to subtle inconsistencies in timing, gaze, or motion, contributing to higher Disturbance scores and lower Animacy and Agency.

The qualitative responses align with the quantitative outcomes observed in the HRIES factors. Participants described the context-aware condition (Orange robot, RC) using terms related to environmental grounding (object referencing and spatial localisation), proactive guidance (gaze/pointing), and social warmth (politeness and friendliness). In contrast, the baseline condition (Blue robot, RN) was frequently described as vague, static, and less socially engaging, with reduced clarity about how to satisfy the robot’s needs. Notably, some participants noted a potential trade-off between explicit guidance and interaction pacing, suggesting that the appropriate level of expressivity may depend on the user profile and scenario. These observations support the interpretation that combining context-sensitive attentional behaviour with expressive cues can increase perceived clarity and Sociability, while also motivating future work on adapting the amount of guidance to the interaction context.

### Limitations

Developing a CABIB system that dynamically updates the robot’s verbal and non-verbal expressiveness brings significant challenges. For this reason, the system’s design, the subsequent implementation in the Mini robot, and the user study introduced limitations that must be disclosed.

On the technical side, one of the most important limitations stems from the design of the biologically inspired system. Whereas it attempts to represent human biology in the robot, the model includes parameters that affect its performance and, therefore, the robot’s final behaviour. Although similar (and also different) versions of this model have been deeply studied in academic papers [19,34], it is an important factor that must be further analysed by conducting sensitivity analysis. The sensitivity analysis and ablation studies can also be applied to analyse the influence of parameter selection on the attention mechanisms and multimodal behaviours such as gaze, pointing, and context-aware speech. While the results show a positive effect of the overall system on user perception, the contribution of each component is not explicitly reviewed.

Another limitation is the generation of the robot’s speech, which could be accomplished with AI. We are aware that LLMs are currently experiencing a trend towards greedy use in technology development. However, we opted to use customisable sentences with keyword replacement to generate an adapted speech. The reason behind this decision is that LLMs require specific computational resources, long inference times for systems that need real-time responses, and usually an internet connection. At present, this is not feasible on a small robotic platform such as the Mini robot. Other design aspects could be addressed using AI, such as environment recognition with Vision Transformer-based perception models or context-awareness with Vision–Language Models. While these models have better perceptual and semantic capabilities, they mostly require high-performance hardware and may require an internet connection.

The user study also has some aspects that warrant comment. First, we selected a video-based evaluation rather than face-to-face interaction. Although some studies [25,82] have followed a similar procedure, video evaluations might yield different results than face-to-face evaluations, due to differences in users’ perceptions or to the diverse reactions users may have compared to a generic video. Additionally, we cannot rule out the possibility that the robot’s colour differences may influence participants’ perceptions, thereby affecting the HRIES questionnaire’s results. Therefore, future studies with face-to-face assessment and with robots of the same colour might be necessary to ensure the validity of the proposed approach and to better control for potential order effects and colour-to-condition confounds. Additionally, the study combined standard questionnaires explicitly designed for human–robot interaction studies with custom questions to elicit participants’ impressions. These are the most appropriate metrics for our study given its aim. However, as this cannot be fully guaranteed, other metrics may complement the results presented in this paper. Furthermore, the robot’s perception identifies the objects in the scene using computer vision. However, we have not considered situations where the robot can find more than one resource of the same type (e.g., two drinks), so the system does not consider priorities based on the user’s (or even the robot’s) preferences for different objects of the same type.

Another limitation of the current study is the nature of the baseline condition used for comparison. The robot without the proposed system lacks contextual awareness and exhibits generic speech and random movements not generated by the Joint Attention System. While this setup allows us to isolate the perceptual impact of the proposed approach, it does not represent stronger alternative baselines, such as rule-based context-aware systems or architectures lacking specific components.

Finally, the implementation of the CABIB system in our Mini robot aims to narrow the gap between humans and our robot’s behaviour. However, deceptive anthropomorphism can lead to the uncanny valley, where robots are perceived as scary. As Mini is intended to work with older people, this matter must be considered when enabling the robot with human features, given users’ vulnerable state. However, in our study, participants were mostly adults without cognitive decline. Hence, their perceptions and expectations of the technology may differ. Therefore, the results may vary if the participants’ ages are closer to the actual Mini users’ age. Future work should test older people’s perception of the CABIB system.

## 7. Conclusions

This paper presents the development and evaluation of a biologically inspired system that generates coherent context-aware verbal and non-verbal expressiveness during human–robot interaction. The system fuses the environmental information captured by the robot’s sensors with the robot’s internal state, which represents its situation and goals. We conducted a user study to assess whether people perceive the context-aware behavioural responses of our Mini robot by comparing two robots with the same appearance, one with and one without the proposed systems. The results show significant statistical differences in the four factors—Sociability, Agency, Animacy, and Disturbance—of the HRIES questionnaire, suggesting that including context-awareness behaviour enhances how people perceive social robots. Additionally, the results highlight that most people could perceive subtle details in the robot’s behaviour, such as the robot looking for the resources it needed or being aware of its surroundings.

Future work in this line of research aims to further extend the robot’s perception and behavioural capabilities during human–robot interaction. Being aware of the environment in which it is operating is essential to being perceived as more natural, capable, and assistive during tasks. For this reason, increasing the robot’s capabilities to assist with additional tasks will require it to perceive and consider a wider range of objects and stimuli in the scene, as well as to improve its speech generation. This could be achieved through AI models, such as Vision–Language Models, which would be explored in future approaches.

In addition, the evaluation of the proposed system will be extended to include stronger baselines, such as rule-based context-aware architectures. Further work will also focus on the design of controlled experiments and ablation studies to analyse the contribution of individual components, including gaze alignment, pointing behaviour, verbal grounding, and internal motivational processes. Finally, we would like to measure whether this kind of behaviour influences users’ actions when completing tasks, which guides their attention towards important environmental resources. In this context, observing users’ responses during joint attention scenarios might be important for a full understanding of the robot’s role in cooperative human–robot interaction.

## Figures and Tables

**Table 1:** Contribution analysis of each system component to the overall software architecture.

Component	Role	Typical Prior Work	Contribution
Biological drive model	Generates internal needs and motivations	Predefined behaviours or externally triggered actions	Introduces endogenous motivation, enabling proactive behaviours such as request generation based on internal state
Decision-Making System	Selects actions based on internal state and context	Rule-based or reactive behaviour selection	Provides goal-directed behaviour driven by internal needs, improving perceived autonomy and coherence
Joint Attention System	Aligns attention with relevant objects and user	Limited or implicit attention mechanisms	Enables shared attention, improving perception of environmental awareness and interaction grounding
Expression Manager	Maps internal states to multimodal expressions	Fixed or loosely coupled expressive behaviours	Ensures consistent and interpretable expression of intentions through coordinated gaze, motion, and actions

**Table 2:** Biological internal processes modelled in the robot, including their range, ideal value, associated deficit name, and variation rate.

Process	Value Range	Ideal Value ($i_{i}$)	Deficit Name	Variation Rate ($r_{i}$)
Feeding	0 to 100	100	Hunger	−0.02
Moisturising	0 to 100	100	Thirst	−0.03
Entertainment	0 to 100	100	Boredom	−0.05

**Table 3:** Robot motivations and associated behaviours, including resource conditions and their functional effects.

Motivation	Behaviour	Resource Status	Definition	Behaviour Effect
Acquire	Request	Resource needed	A biological deficit exceeds a predefined threshold, and the required object is not available.	The robot verbally requests the object needed to regulate its internal process.
Consume	Accept-Eat, drink, play	Resource received and needed	The user provides an object that matches the internal deficit.	The robot takes the object to play, eat, or drink.
Avoid	Reject	Resource received and not needed	The user provides an object that does not correspond to the active deficit.	The robot verbally rejects the given object.

**Table 4:** Saliency weights ($\lambda_{i}$) assigned to each stimulus based on their contextual relevance.

Detector	Head Pose	Face Detector	Object Detector
Saliency weight $\lambda_{i}$	1.0	0.7	0.5

**Table 5:** Wilcoxon signed-rank test results comparing Blue and Orange robot conditions for each HRIES factor. Medians and interquartile ranges (IQR) are reported. Effect size *r* is calculated as $r=Z/\sqrt{N}$. Significance is shown after Bonferroni correction (α=0.0125). The number of asterisks (*) indicate the level of statistical significance: “***” indicates very significant $p_{\mathrm{value}}<0.001$ and “*” indicates subtly significant $p_{\mathrm{value}}<0.05$.

Factor	Robot	Median (IQR)	Mean ± SD	*Z*	*p*	*r*	Bonferroni Correction
Sociability	Blue (RN)	3.50 (2.25–4.75)	3.64 ± 1.57	−7.84	<0.001 ***	0.68	Yes
	Orange (RC)	5.50 (4.50–6.50)	5.28 ± 1.39				
Agency	Blue (RN)	2.75 (2.00–4.25)	3.11 ± 1.47	−6.20	<0.001 ***	0.54	Yes
	Orange (RC)	4.00 (2.50–5.00)	3.87 ± 1.54				
Animacy	Blue (RN)	3.75 (3.00–4.75)	3.80 ± 1.31	−7.96	<0.001 ***	0.69	Yes
	Orange (RC)	5.00 (4.00–6.00)	4.91 ± 1.32				
Disturbance	Blue (RN)	2.50 (1.50–3.50)	2.72 ± 1.35	2.82	0.005 *	0.24	Yes
	Orange (RC)	2.25 (1.50–3.25)	2.44 ± 1.10				

**Table 6:** Distribution of participants’ responses to the questions. (a) Have you noticed any of the robots could perceive their surroundings? (b) Have you felt that any of the robots looked at or pointed to the objects they wanted or needed? and (c) Have you felt that any of the robots is aware of its own state and environment?

Question	Orange Only	Both	Blue Only	None
Perceive surroundings	96 (72%)	20 (15%)	6 (5%)	10 (8%)
Look/point to objects	88 (66%)	28 (21%)	6 (3%)	11 (8%)
Aware of state and environment	108 (81%)	9 (7%)	12 (9%)	4 (<3%)

## Data Availability

Data is available upon request.

## References

[1] Xu Q., Ng J., Tan O., Huang Z., Tay B., Park T. (2015). Methodological issues in scenario-based evaluation of human–robot interaction. Int. J. Soc. Robot..

[2] Khairy D., Abougalala R.A., Areed M.F., Atawy S.M., Alkhalaf S., Amasha M. (2020). Educational robotics based on artificial intelligence and context-awareness technology: A framework. J. Theor. Appl. Inf. Technol..

[3] Belpaeme T., Kennedy J., Ramachandran A., Scassellati B., Tanaka F. (2018). Social robots for education: A review. Sci. Robot..

[4] Ahmad M.I., Mubin O., Orlando J. (2016). Children views’ on social robot’s adaptations in education. Proceedings of the 28th Australian Conference on Computer-Human Interaction.

[5] MD H.A. (2025). Meta-learning approaches for causal discovery in dynamic healthcare and robotics environments. Mesopotamian J. Artif. Intell. Healthc..

[6] Kubota A., Riek L.D. (2022). Methods for robot behavior adaptation for cognitive neurorehabilitation. Annu. Rev. Control. Robot. Auton. Syst..

[7] Morgan A.A., Abdi J., Syed M.A., Kohen G.E., Barlow P., Vizcaychipi M.P. (2022). Robots in healthcare: A scoping review. Curr. Robot. Rep..

[8] Wijayathunga L., Rassau A., Chai D. (2023). Challenges and solutions for autonomous ground robot scene understanding and navigation in unstructured outdoor environments: A review. Appl. Sci..

[9] Billings D.R., Schaefer K.E., Chen J.Y., Hancock P.A. (2012). Human-robot interaction: Developing trust in robots. Proceedings of the Seventh Annual ACM/IEEE International Conference on Human-Robot Interaction.

[10] García-Martínez J., Gamboa-Montero J.J., Castillo J.C., Castro-González Á. (2024). Analyzing the Impact of Responding to Joint Attention on the User Perception of the Robot in Human-Robot Interaction. Biomimetics.

[11] Blakemore S.J., Decety J. (2001). From the perception of action to the understanding of intention. Nat. Rev. Neurosci..

[12] Jung H., Park S., Joe S., Woo S., Choi W., Bae W. (2025). AI-Driven Control Strategies for Biomimetic Robotics: Trends, Challenges, and Future Directions. Biomimetics.

[13] Duncan J.A., Alambeigi F., Pryor M.W. (2024). A survey of multimodal perception methods for human–robot interaction in social environments. ACM Trans.-Hum.-Robot. Interact..

[14] García-Martínez J., Gamboa-Montero J.J., Castillo J.C., Castro-González Á., Salichs M.A. (2025). Implementation of a Biologically Inspired Responsive Joint Attention System for a Social Robot. Adv. Intell. Syst..

[15] De Graaf M.M., Allouch S.B. (2013). Exploring influencing variables for the acceptance of social robots. Robot. Auton. Syst..

[16] Naneva S., Sarda Gou M., Webb T.L., Prescott T.J. (2020). A systematic review of attitudes, anxiety, acceptance, and trust towards social robots. Int. J. Soc. Robot..

[17] Fernández-Rodicio E., Gamboa-Montero J.J., Maroto-Gómez M., Castro-González Á., Salichs M.A. (2025). Evaluating the effect of co-speech gesture prediction on human–robot interaction. Int. J. Hum. Comput. Stud..

[18] Urrea C. (2025). Artificial intelligence-driven and bio-inspired control strategies for industrial robotics: A systematic review of trends, challenges, and sustainable innovations toward industry 5.0. Machines.

[19] Maroto-Gómez M., Malfaz M., Castro-Gonzalez A., Salichs M.A. (2023). A motivational model based on artificial biological functions for the intelligent decision-making of social robots. Memetic Comput..

[20] Salichs M.A., Castro-González Á., Salichs E., Fernández-Rodicio E., Maroto-Gómez M., Gamboa-Montero J.J., Marques-Villarroya S., Castillo J.C., Alonso-Martín F., Malfaz M. (2020). Mini: A new social robot for the elderly. Int. J. Soc. Robot..

[21] Spatola N., Kühnlenz B., Cheng G. (2021). Perception and evaluation in human–robot interaction: The Human–Robot Interaction Evaluation Scale (HRIES)—A multicomponent approach of anthropomorphism. Int. J. Soc. Robot..

[22] Desimone R., Duncan J. (1995). Neural mechanisms of selective visual attention. Annu. Rev. Neurosci..

[23] Premebida C., Ambrus R., Marton Z.C. (2018). Intelligent robotic perception systems. Applications of Mobile Robots.

[24] Li J., Xu Z., Zhu D., Dong K., Yan T., Zeng Z., Yang S.X. (2021). Bio-inspired intelligence with applications to robotics: A survey. Intell. Robot..

[25] Wang J., Lin S., Liu A. (2023). Bioinspired perception and navigation of service robots in indoor environments: A review. Biomimetics.

[26] Zaraki A., Pieroni M., De Rossi D., Mazzei D., Garofalo R., Cominelli L., Dehkordi M.B. (2016). Design and evaluation of a unique social perception system for human–robot interaction. IEEE Trans. Cogn. Dev. Syst..

[27] Mascaro R., Chli M. (2025). Scene representations for robotic spatial perception. Annu. Rev. Control. Robot. Auton. Syst..

[28] Marques-Villarroya S., Castillo J.C., Fernández-Rodicio E., Salichs M.A. (2024). A bio-inspired exogenous attention-based architecture for social robots. Expert Syst. Appl..

[29] Marques-Villarroya S., Castillo J.C., Gamboa-Montero J.J., Sevilla-Salcedo J., Salichs M.A. (2022). A bio-inspired endogenous attention-based architecture for a social robot. Sensors.

[30] Yi Y., Zhuo W., Huifeng W., Danfeng S., Simon R. (2026). Bio-inspired unified model for representing geometric relations in robotic perception. Sci. Rep..

[31] Velsquez J. (1997). Modeling emotions and other motivations in synthetic agents. Proceedings of the Fourteenth National Conference on Artificial Intelligence.

[32] Canamero D. (1997). Modeling motivations and emotions as a basis for intelligent behavior. Proceedings of the First International Conference on Autonomous Agents.

[33] De Oliveira R.F., Damisch L., Hossner E.J., Oudejans R.R., Raab M., Volz K.G., Williams A.M. (2009). The bidirectional links between decision making, perception, and action. Prog. Brain Res..

[34] Maroto-Gómez M., Castro-González Á., Malfaz M., Salichs M.Á. (2023). A biologically inspired decision-making system for the autonomous adaptive behavior of social robots. Complex Intell. Syst..

[35] Lapointe R. (2025). Toward a Reflective Motivational Architecture for Artificial Intelligence: Instinct-Driven Evaluation and Emotion in Synthetic Agents. https://ssrn.com/abstract=5297948.

[36] Aliasghari P., Ghafurian M., Nehaniv C.L., Dautenhahn K. (2025). A Biologically Inspired Program-Level Imitation Approach for Robots. IEEE Access.

[37] Chen R., Minato T., Sakai K., Kanda T. (2025). Meet the Motivational Robot That Predicts Your Future Feelings. ACM Trans.-Hum.-Robot. Interact..

[38] Moore C., Dunham P.J., Dunham P. (2014). Joint Attention: Its Origins and Role in Development.

[39] Tomasello M. (2014). Joint attention as social cognition. Joint Attention.

[40] Murphy R.R., Nomura T., Billard A., Burke J.L. (2010). Human–robot interaction. IEEE Robot. Autom. Mag..

[41] Kaplan F., Hafner V.V. (2006). The challenges of joint attention. Interact. Stud..

[42] Fiore S.M., Wiltshire T.J., Lobato E.J., Jentsch F.G., Huang W.H., Axelrod B. (2013). Toward understanding social cues and signals in human–robot interaction: Effects of robot gaze and proxemic behavior. Front. Psychol..

[43] Itti L., Koch C., Niebur E. (2002). A model of saliency-based visual attention for rapid scene analysis. IEEE Trans. Pattern Anal. Mach. Intell..

[44] Drelie Gelasca E., Tomasic D., Ebrahimi T. Which colors best catch your eyes: A subjective study of color saliency. Proceedings of the First International Workshop on Video Processing and Quality Metrics for Consumer Electronics.

[45] Yantis S., Jonides J. (1984). Abrupt visual onsets and selective attention: Evidence from visual search. J. Exp. Psychol. Hum. Percept. Perform..

[46] Mundy P., Gomes A. (1998). Individual differences in joint attention skill development in the second year. Infant Behav. Dev..

[47] Scaife M., Bruner J.S. (1975). The capacity for joint visual attention in the infant. Nature.

[48] Breazeal C., Kidd C.D., Thomaz A.L., Hoffman G., Berlin M. (2005). Effects of nonverbal communication on efficiency and robustness in human-robot teamwork. Proceedings of the 2005 IEEE/RSJ International Conference on Intelligent Robots and Systems.

[49] Pereira A., Oertel C., Fermoselle L., Mendelson J., Gustafson J. (2019). Responsive joint attention in human-robot interaction. Proceedings of the 2019 IEEE/RSJ International Conference on Intelligent Robots and Systems (IROS).

[50] Mishra C., Skantze G. (2022). Knowing where to look: A planning-based architecture to automate the gaze behavior of social robots. Proceedings of the 2022 31st IEEE International Conference on Robot and Human Interactive Communication (RO-MAN).

[51] Huang C.M., Thomaz A.L. (2011). Effects of responding to, initiating and ensuring joint attention in human-robot interaction. Proceedings of the 2011 Ro-Man.

[52] Leekam S.R., Ramsden C.A. (2006). Dyadic orienting and joint attention in preschool children with autism. J. Autism Dev. Disord..

[53] Brennan S.E., Chen X., Dickinson C.A., Neider M.B., Zelinsky G.J. (2008). Coordinating cognition: The costs and benefits of shared gaze during collaborative search. Cognition.

[54] Hobson R.P. (2005). What puts the jointness into joint attention. Joint Attention: Communication and Other Minds.

[55] Sani-Bozkurt S., Bozkus-Genc G. (2023). Social robots for joint attention development in autism spectrum disorder: A systematic review. Int. J. Disabil. Dev. Educ..

[56] Meltzoff A.N., Brooks R., Shon A.P., Rao R.P. (2010). “Social” robots are psychological agents for infants: A test of gaze following. Neural Netw..

[57] Anzalone S.M., Tilmont E., Boucenna S., Xavier J., Jouen A.L., Bodeau N., Maharatna K., Chetouani M., Cohen D., Group M.S. (2014). How children with autism spectrum disorder behave and explore the 4-dimensional (spatial 3D+ time) environment during a joint attention induction task with a robot. Res. Autism Spectr. Disord..

[58] Zheng Z., Zhao H., Swanson A.R., Weitlauf A.S., Warren Z.E., Sarkar N. (2017). Design, development, and evaluation of a noninvasive autonomous robot-mediated joint attention intervention system for young children with ASD. IEEE Trans.-Hum.-Mach. Syst..

[59] Scassellati B., Boccanfuso L., Huang C.M., Mademtzi M., Qin M., Salomons N., Ventola P., Shic F. (2018). Improving social skills in children with ASD using a long-term, in-home social robot. Sci. Robot..

[60] Naendrup-Poell L., Onnasch L. (2025). Predictive robot eyes enhance attentional guidance in cooperative human–robot interaction. Sci. Rep..

[61] Lavit Nicora M., Prajod P., Mondellini M., Tauro G., Vertechy R., André E., Malosio M. (2024). Gaze detection as a social cue to initiate natural human-robot collaboration in an assembly task. Front. Robot. AI.

[62] Prajod P., Nicora M.L., Mondellini M., Tauro G., Vertechy R., Malosio M., André E. (2023). Gaze detection and analysis for initiating joint activity in industrial human-robot collaboration. arXiv.

[63] Prajod P., Lavit Nicora M., Malosio M., André E. (2023). Gaze-based attention recognition for human-robot collaboration. Proceedings of the 16th International Conference on PErvasive Technologies Related to Assistive Environments.

[64] Cazzato D., Mazzeo P.L., Spagnolo P., Distante C. (2015). Automatic joint attention detection during interaction with a humanoid robot. Proceedings of the International Conference on Social Robotics.

[65] Morillo-Mendez L., Stower R., Sleat A., Schreiter T., Leite I., Mozos O.M., Schrooten M.G. (2023). Can the robot “see” what I see? Robot gaze drives attention depending on mental state attribution. Front. Psychol..

[66] García-Martínez J., Gamboa-Montero J.J., Castillo J.C., Castro-González Á., Salichs M.Á. (2024). Bio-Inspired Joint Attention System for Dynamic Focus of Attention Allocation and Real-Time Stimulus Prioritization in Social Robots. Proceedings of the International Conference on Social Robotics.

[67] García-Martínez J., Gamboa-Montero J.J., Castro-González Á., Castillo J.C. (2025). Are Robots More Engaging When They Respond to Joint Attention? Findings from a Turn-Taking Game with a Social Robot. Appl. Sci..

[68] Hochachka P.W., Somero G.N. (2002). Biochemical Adaptation: Mechanism and Process in Physiological Evolution.

[69] Ryan R.M., Bradshaw E., Deci E.L., Sternberg R., Pickren W. (2019). A history of human motivation theories. The Cambridge Handbook of the Intellectual History of Psychology.

[70] Cutsuridis V., Hussain A., Taylor J.G. (2011). Perception-Action Cycle: Models, Architectures, and Hardware.

[71] Cutsuridis V. (2013). Cognitive models of the perception-action cycle: A view from the brain. Proceedings of the 2013 International Joint Conference on Neural Networks (IJCNN).

[72] Frischen A., Bayliss A.P., Tipper S.P. (2007). Gaze cueing of attention: Visual attention, social cognition, and individual differences. Psychol. Bull..

[73] Ferreira J.F., Dias J. (2014). Attentional mechanisms for socially interactive robots—A survey. IEEE Trans. Auton. Ment. Dev..

[74] Wertheimer M. (1938). Gestalt Theory.

[75] Posner M. (1984). Components of visual orienting. Attention and Performance X: Control of Language Processes.

[76] Day B.L., Fitzpatrick R.C. (2005). The vestibular system. Curr. Biol..

[77] Rosenthal R., Cooper H., Hedges L. (1994). Parametric measures of effect size. Handb. Res. Synth..

[78] Braun V., Clarke V. (2006). Using thematic analysis in psychology. Qual. Res. Psychol..

[79] Fink J. (2012). Anthropomorphism and human likeness in the design of robots and human-robot interaction. Proceedings of the International Conference on Social Robotics.

[80] Maroto-Gómez M., Fernández-Rodicio E., Castro-González Á., Malfaz M., Salichs M.Á. (2024). Evaluating Users’ Perception of Biologically Inspired Involuntary Behavior in Human–Robot Interaction. Adv. Intell. Syst..

[81] Maroto-Gómez M., Álvarez-Arias S., Rodríguez-Huelves J., Segura-Bencomo A., Malfaz M. (2025). A robot companion with adaptive object preferences and emotional responses enhances naturalness in human–robot interaction. Electronics.

[82] Rossi S., Coppola A., Gaita M., Rossi A. (2023). Human–Robot Interaction Video Sequencing Task (HRIVST) for Robot’s Behavior Legibility. IEEE Trans.-Hum.-Mach. Syst..

